# Pathology of the conus medullaris and cauda equina. Beyond the usual suspects

**DOI:** 10.1186/s13244-025-02117-z

**Published:** 2025-10-25

**Authors:** Nerses Nersesyan, Maria Lucia Brun Vergara, Azza Reda, Suely Fazio Ferraciolli, Leandro Lucato, Carlos Torres

**Affiliations:** 1 The Ottawa Hospital, University of Ottawa, Ottawa, Canada; 2 Hospital Josep Trueta, University of Girona, Girona, Spain; 3 King Abdulaziz University, Jeddah, Saudi Arabia; 4 Pediatric Imaging Research Center, Harvard Medical School & Massachusetts General Hospital, Boston, MA USA; 5 Hospital das Clínicas, Faculty of Medicine, University of São Paulo, São Paulo, Brazil

**Keywords:** Conus medullaris, Cauda equina, Primary conus medullaris glioma, Spinal schistosomiasis, MOGAD

## Abstract

**Background:**

Pathologies affecting the conus medullaris and cauda equina can present with overlapping clinical symptoms, making an accurate diagnosis essential. Conus medullaris syndrome results from damage at the T12–L2 level, while cauda equina syndrome arises from nerve root compression below the conus. Both conditions may cause motor deficits, sensory disturbances, and autonomic dysfunction, necessitating a detailed differential diagnosis.

**Objective:**

This educational review highlights common and rare etiologies of conus medullaris and cauda equina lesions, emphasizing imaging characteristics and diagnostic considerations. A comprehensive review of tumors, infections, inflammatory, vascular, and degenerative conditions affecting these regions was performed. Contrast-enhanced MRI was identified as the gold standard for diagnosis.

**Revised pathologies:**

- Tumors: myxopapillary ependymomas and schwannomas are the most frequent neoplasms, while drop metastases and glioblastomas represent rarer entities.
- Infections: tuberculous arachnoiditis, bacterial radiculitis, schistosomiasis, and neurocysticercosis may mimic neoplastic processes.
- Inflammatory disorders: Guillain–Barré syndrome, neurosarcoidosis, and MOGAD may cause nerve root thickening and enhancement.
- Vascular lesions: spinal dural arteriovenous fistulas, infarcts, and arteriovenous malformations can produce conus and cauda equina symptoms.
- Miscellaneous causes: developmental anomalies like diastematomyelia and ventriculus terminalis, along with degenerative diseases, can mimic other conditions.

**Conclusion:**

Radiologists play a pivotal role in differentiating conus medullaris and cauda equina pathologies. A thorough understanding of imaging findings is essential for accurate diagnosis and effective management.

**Critical relevance statement:**

Conus medullaris and cauda lesions present with overlapping clinical symptoms but show some distinct imaging patterns. It is essential to recognize characteristic features that differentiate neoplastic from infectious or vascular etiologies.

**Key Points:**

- Conus and cauda lesions have varied causes; MRI with contrast is vital for accurate diagnosis.
- Myxopapillary ependymomas cause vertebral scalloping; schwannomas may be cystic; intramedullary gliomas expand the cord.
- Conus medullaris and cauda lesions overlap clinically; imaging helps distinguish neoplastic from infectious or vascular causes.

**Graphical Abstract:**

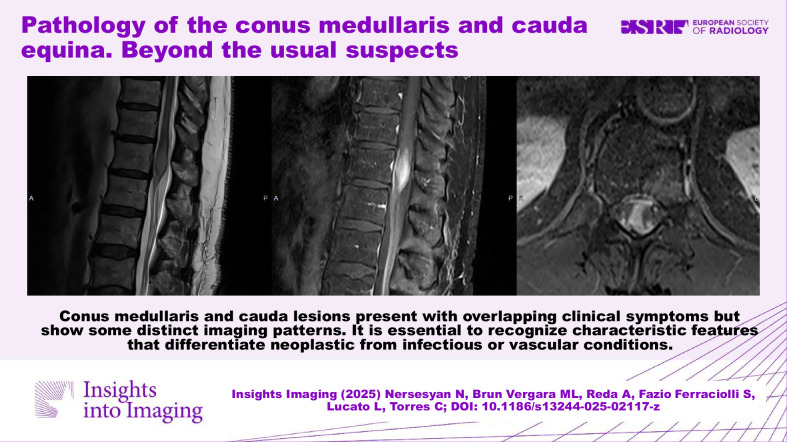

## Introduction

Disorders affecting the conus medullaris and the nerve roots of the cauda equina often produce overlapping clinical symptoms, making differentiation challenging [1]. The conus medullaris, forming the terminal segment of the spinal cord, typically resides at the T12–L1 vertebral level. Injury or disease at this junction-particularly between T12 and L2-can result in conus medullaris syndrome, which is characterized by a combination of upper and lower motor neuron deficits due to involvement of both the spinal cord and adjacent nerve roots [2].

In contrast, cauda equina syndrome arises from damage or compression of the nerve roots below the conus, most commonly due to acute herniation of a lumbar intervertebral disc, but also from other causes such as trauma, tumors, or infections. This syndrome is considered a neurological emergency and, while rare, has an estimated incidence of about 1 in 65,000 individuals [1, 3].

The clinical manifestations of cauda equina syndrome can develop suddenly or progress over time, and diagnosis generally requires the presence of at least two hallmark features: sensory disturbances in the perianal or “saddle” region, and dysfunction of bowel, bladder, or sexual function. Additional symptoms may include low back pain, radiating leg pain, lower limb weakness, diminished reflexes, and sensory changes in the legs [1, 3, 4].

Patients with conus medullaris syndrome may similarly present with severe back pain, saddle anesthesia, urinary retention, bowel incontinence, and lower limb weakness. However, a distinguishing feature is the simultaneous presence of upper and lower motor neuron signs, whereas cauda equina syndrome results exclusively in lower motor neuron deficits [1, 2, 5].

Given the clinical overlap and the potential for systemic conditions to affect both the conus medullaris and cauda equina, radiologists play a crucial role in identifying the underlying etiology using advanced imaging, particularly MRI. Some pathologies may present with atypical or widespread symptoms, further complicating the clinical picture [3, 4].

This review aims to outline the most frequent and important causes to consider when evaluating lesions in the conus medullaris and cauda equina regions, as well as to highlight less common entities that should be included in the differential diagnosis. The goal is to enhance diagnostic accuracy and guide appropriate management by recognizing both typical and unusual imaging patterns.

## Recommended imaging modalities and protocol

The most important imaging modality for diagnosing lesions of the conus medullaris and cauda equina is magnetic resonance imaging (MRI), as it provides superior soft tissue contrast, detailed anatomic resolution, and direct visualization of the spinal cord, nerve roots, and associated pathology [6]. MRI is considered the gold standard for both syndromes, far surpassing other modalities such as CT, which is primarily of value when MRI is contraindicated or to assess bony integrity [6, 7]. The recommended MRI protocol for evaluating the conus medullaris and cauda equina should include mandatory sequences: sagittal and axial T1-weighted (T1W) images, sagittal and axial T2-weighted (T2W) images, and a sagittal STIR (short tau inversion recovery) or fat-suppressed T2 sequence. These sequences are critical for identifying compressive or infiltrative lesions, inflammatory changes, and assessing nerve root involvement [6–8]. Optional sequences include postcontrast (gadolinium-enhanced) T1W images are indicated if neoplasm, infection, or inflammatory etiology is suspected. Advanced sequences like T2-SPACE or diffusion imaging are helpful for improved nerve root characterization and spinal pathology assessment [6–8].

## Tumor

### The usual suspects

#### Myxopapillary ependymoma

Myxopapillary ependymomas are a type of ependymoma that mainly occur in the filum terminale and conus medullaris [9–11].

They are one of the most common tumors in the cauda equina region and are primarily intradural and extramedullary spinal tumors that occur in the lumbosacral spine [9, 11–13]. Rarely, they can arise in the cervicothoracic spine or fourth ventricle [12].

**Fig. 1:**
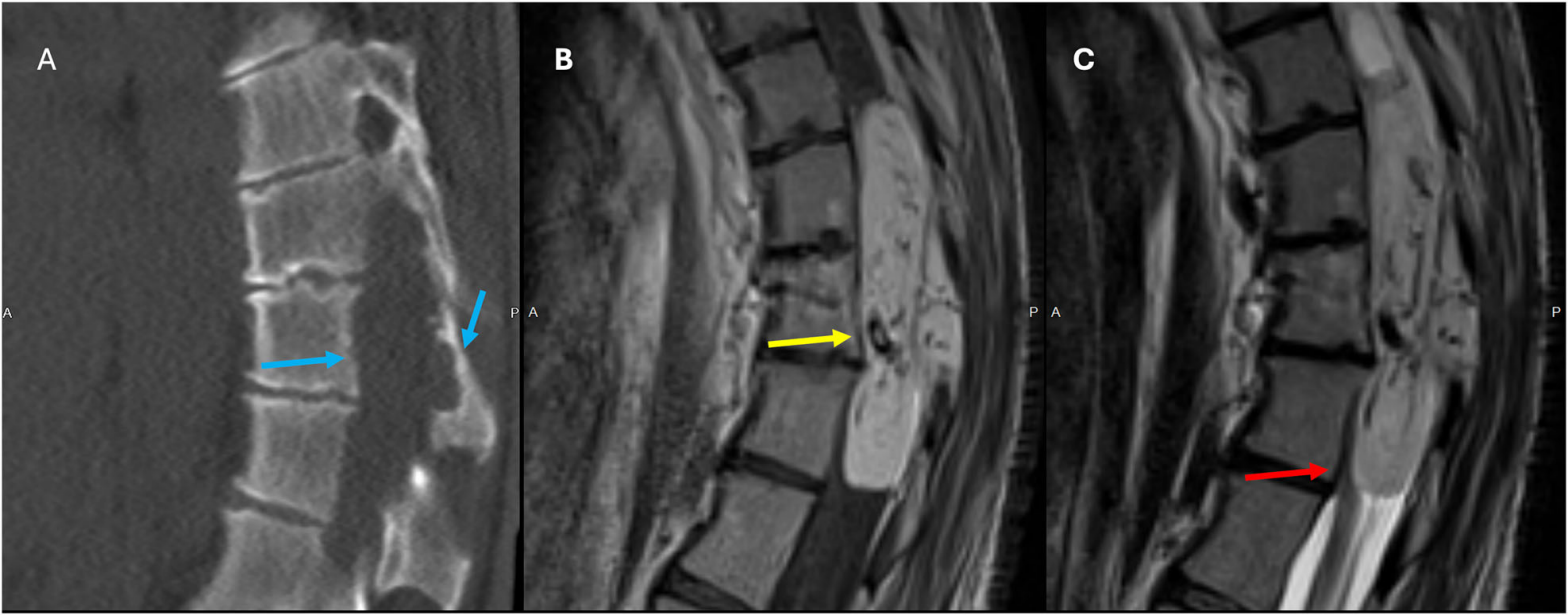
36-year-old patient with a history of cerebral palsy, admitted for urinary retention and sepsis. **A** Unenhanced CT of the spine showing an isodense expansile mass lesion with posterior vertebral body and posterior elements scalloping (blue arrows). T1 postcontrast (**B** image), T2W (**C** image) images showing a large well-defined smooth, expansile mixed signal, heterogeneously enhancing intradural, extramedullary mass arising from the filum terminale and extending from T11 to L2 (yellow arrow). This is truncating the conus and obliterating the central canal with cauda equina compression (red arrow)

On imaging, myxopapillary ependymomas are well-defined tumors that can be sausage-shaped and span more than one vertebral level, where they can cause scalloping of the vertebral bodies (Fig. 1A) [10, 11]. They usually show isointensity on T1W and overall high intensity on T2W sequences (Fig. 1C). As more atypical findings, low signal intensity may be seen at the tumor margins due to hemorrhage, and calcification may lead to regions of low T2 signal. The enhancement pattern is typically homogeneous and virtually always seen on T1W sequences postcontrast (Fig. 1B) [9–12].

They are generally slow-growing, but they are considered World Health Organization (WHO) grade 2 tumors that can sometimes metastasize [11, 14]. Surgical excision is often successful, and the prognosis is excellent, with a 5-year survival rate over 98%. Some locally aggressive sacral lesions metastasize to lymph nodes, lungs, and bone [14]. Aggressive behavior is more commonly seen in children [9].

#### Cystic schwannoma

Schwannomas are benign nerve sheath tumors in the spinal canal that arise from nerve roots [12]. They are the most common nerve sheath tumors of the spine [10, 12, 15]. Schwannomas typically appear as solid, well-defined masses and are usually intradural extramedullary in location [16].

Schwannomas have a greater incidence in people between the fifth and seventh decades of life. Most spinal schwannomas are solitary and sporadic (95%) [10, 12, 13]. However, they are sometimes associated with neurofibromatosis type 2 (NF2) [12].

**Fig. 2:**
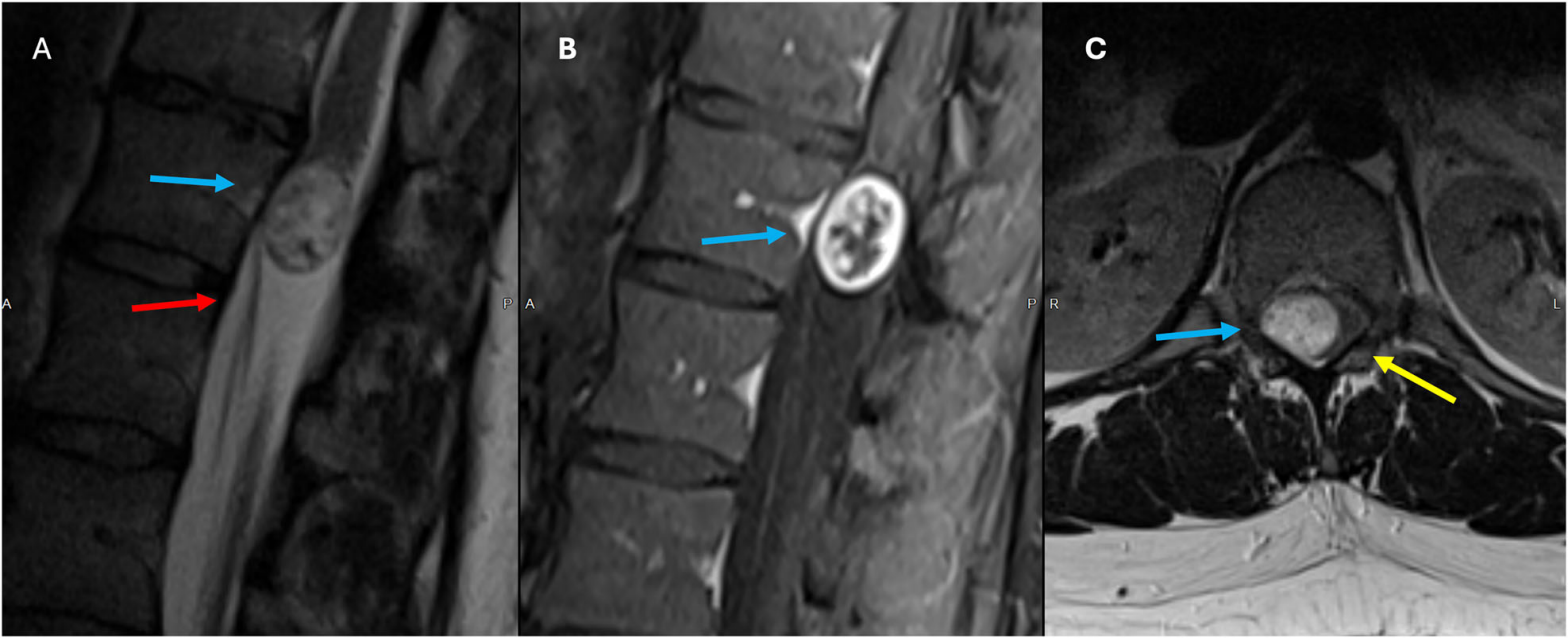
Seventy-year-old male patient with an incidentally identified mass in routine Abdominal CT (not shown). Sagittal and axial T2W (**A**, **C**) and Sagittal T1 Fatsat postcontrast (**B**) images showing an uniloculated cystic intradural extramedullary lesion at T11–L1 levels (blue arrows). The lesion is anteriorly displacing the cauda equina (red arrow) and compressing the conus towards the left (yellow arrow)

MRI is the imaging modality of choice to identify Schwannomas, which usually appear as solid, well-defined, rounded sausage-shaped masses, often with associated adjacent bony remodeling [15, 17, 18]. Although they can appear identical to neurofibromas and meningiomas, schwannomas are frequently associated with hemorrhage, intrinsic vascular changes (thrombosis, sinusoidal dilatation), cyst formation (Fig. 2), and fatty degeneration [19]. These findings are rare in neurofibromas. Schwannomas are usually isointense on T1 (75%), hyperintense on T2 (Fig. 2A, C), often with mixed signal (95%), and demonstrate postcontrast enhancement in virtually 100% (Fig. 2B). Melanotic schwannomas are an exception to the above signal characteristics, as they show high T1 and low T2 signal, given their content of melanine [16, 17, 20].

Surgery is the preferred treatment for these slow-growing tumors, and gross total resection is usually curative for patients with sporadic tumors [13, 15, 20].

#### Leptomeningeal metastases (LM)

LMs are a complication that can occur in the later stages of systemic malignancies [21]. They affect around 5–10% of patients with solid and hematologic neoplasms and can lead to the development of new neurological deficits [21–23].

The most common solid tumors causing LM are lung cancer (particularly non-small cell lung cancer with EGFR mutations and ALK rearrangements), breast cancer (especially HER2-positive and triple-negative subtypes), and melanoma [23, 24]. Additional important causes include renal cell carcinoma, gastrointestinal cancers, and hematologic malignancies such as acute lymphoblastic leukemia and non-Hodgkin lymphoma. These malignancies demonstrate varying propensities for leptomeningeal involvement, with melanoma and lung adenocarcinoma showing the highest involvement [25].

These metastases can be detected with contrast-enhanced MRI follow-ups, which show nodular or diffuse meningeal enhancement (Fig. S1) [21]. High-volume cerebrospinal fluid (CSF) taps can then confirm their presence [21, 26]. While there are several systemic therapeutic options available, the main goal of treatment is palliation, as the mean survival time ranges from 3–6 months [23].

### Beyond the usual

#### Drop metastases

Drop metastases refer to the spread of a primary brain tumor through the subarachnoid space, resulting in intradural extramedullary metastatic lesions of the spine [23, 26, 27]. They are located inside the dura but outside the spinal cord, and usually originate from brain tumors such as pineal tumors, ependymomas (Fig. 4), medulloblastomas, primitive neuroectodermal tumors (PNET), and high-grade gliomas [28].

Medulloblastoma is the most common source, accounting for half of the patients with drop metastases. It is typically diagnosed in children under 10 years of age, with a smaller peak incidence between 15 years and 35 years of age [9, 28]. Glioblastoma is the second most common tumor, occurring in 1% of cases and in 15% of all patients with drop metastases [23, 26, 27].

In children, the most common tumors that lead to drop metastases are medulloblastomas, ependymomas, germinomas, and pineoblastomas, while choroid plexus neoplasms and teratomas are less common [9, 28]. Germinomas and pineoblastomas are the two most common types of pineal tumors that cause drop metastases [29].

**Fig. 3:**
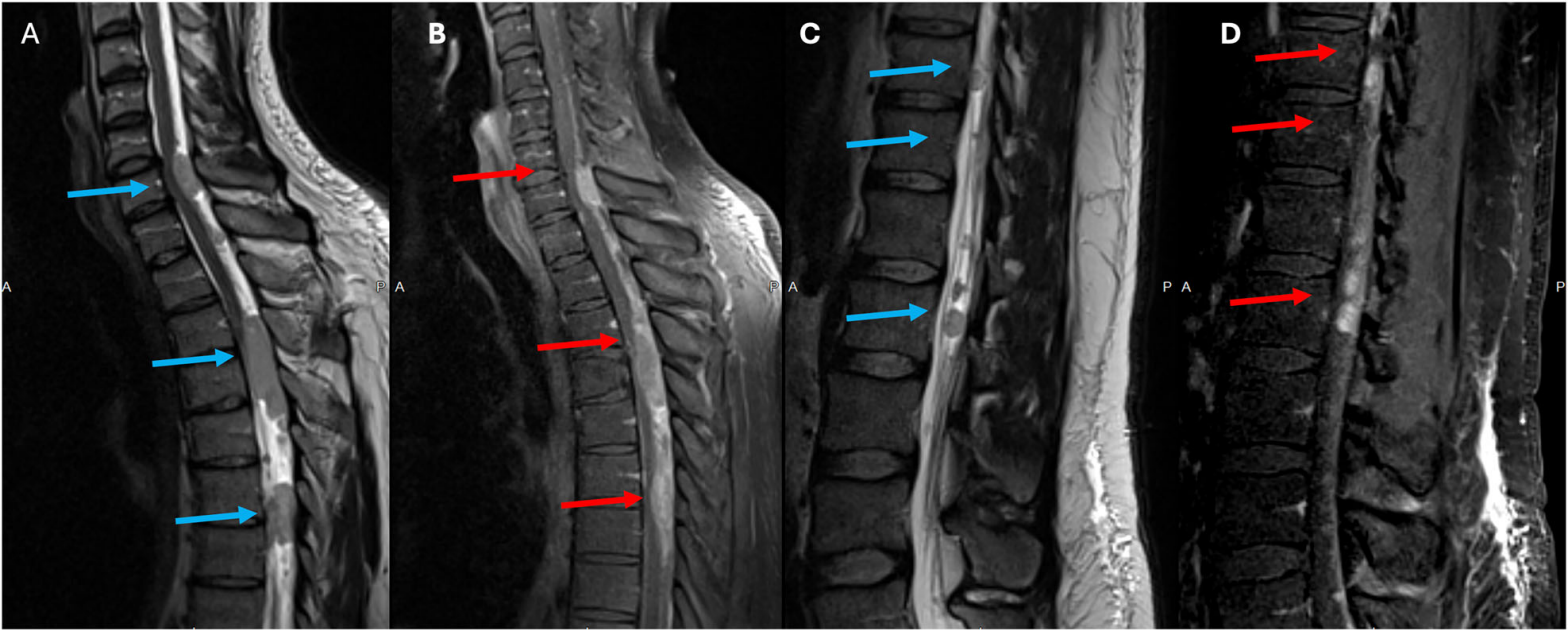
Fifty-four-year-old male patient with headaches and gait disturbances. Whole spine MRI with T2 (**A**, **C**) T1 postcontrast images (**B**, **D**) showing T2 well-defined, confluent, T2 isointense (blue arrows) and homogeneously enhancing intradural lesions (red arrows) starting from C7 and extending caudally towards L1. The lesions are displacing anteriorly and compressing the spinal cord

Ependymomas are a type of CNS tumor that make up 3–6% of all cases [30]. Almost 50% of ependymomas originate from the spinal cord and are the most common type of primary intramedullary spinal cord tumors, accounting for 30–45% of these cases [30–32]. Higher-grade tumors are more likely to cause drop metastases and leptomeningeal spread of disease (Fig. 3) [31].

#### Conus intramedullary metastases

Intramedullary spinal cord metastasis (ISCM) is a rare clinical condition that accounts for only 0.1–0.4% of all patients with malignant lesions and 4–8.5% of central nervous system (CNS) metastases [33].

Lung malignancies are the most common primary source, accounting for approximately 40% of reported cases [24]. Adenocarcinoma of the lung demonstrates a particular predilection for intramedullary spinal cord seeding, including the conus medullaris region [34]. Breast adenocarcinoma constitutes the second most frequent primary tumor, representing approximately 20% of conus medullaris metastases [34]. Other malignancies include renal cell carcinoma, prostate adenocarcinoma, and malignant melanoma. Less commonly reported primary sources include ovarian carcinoma, thyroid carcinoma, pancreatic adenocarcinoma, endometrial adenocarcinoma, and colorectal carcinoma [24, 34, 35].

MRI of the whole spine will reveal an enhancing lesion within the conus medullaris with associated surrounding edema [33, 36].

#### Primary conus medullaris glioma

Primary spinal glioblastoma multiforme (GBM) is an extremely malignant tumor [37, 38]. The median survival rate for patients diagnosed with this condition, even after comprehensive treatment, is only 14 months [38].

**Fig. 4:**
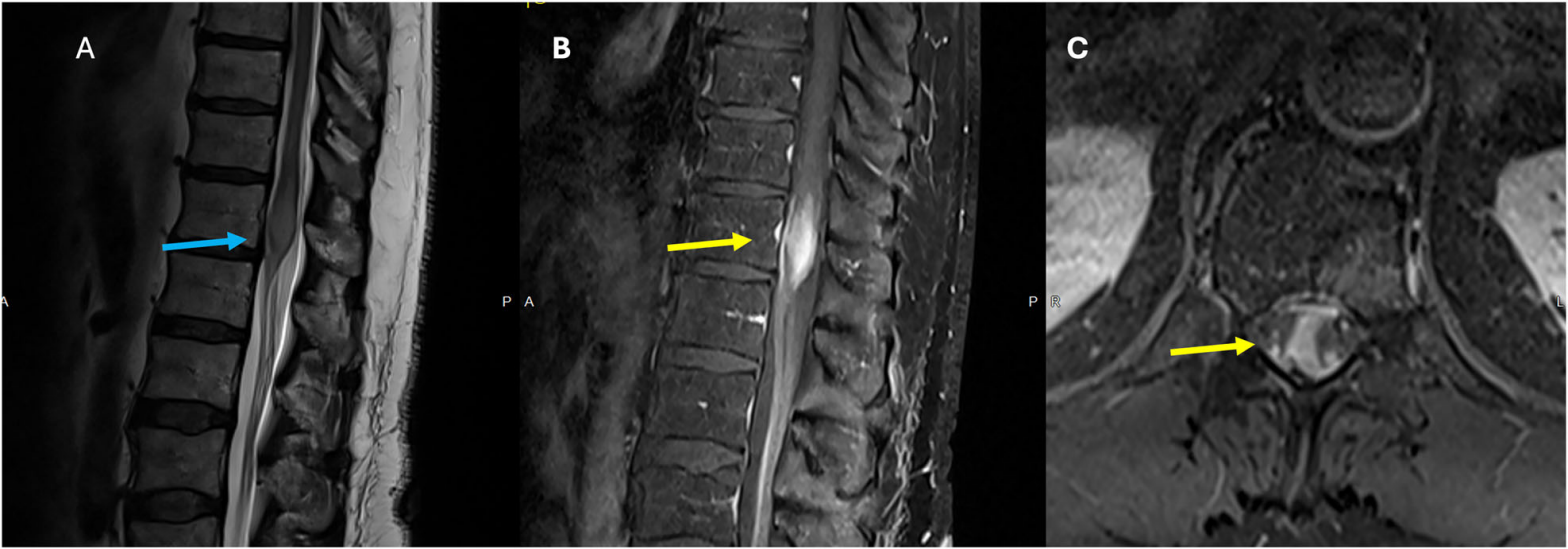
Fifty-six-year-old male presenting with low back pain, lower extremity numbness, and weakness. **A** T2 Sagittal image shows a hyperintense lesion in the conus, with significant cord expansion (blue arrows). **B** T1 Fatsat postcontrast sagittal (**B**) and axial (**C**) images show prominent enhancement of the expanded conus

Primary spinal GBM usually develops in young adults (26–40 years), involving predominantly the cervical and thoracic cord, and is rare in the conus medullaris region (Fig. 4) [37, 38]. The initial imaging method recognized for diagnosing intramedullary tumors is MRI, which may show heterogeneous enhancement and cord expansion. Gadolinium-enhanced MRI throughout the entire neuroaxis is highly advised to exclude metastasis, evaluate treatment effectiveness, and detect possible recurrence [38].

## Infection

### Usual suspects

#### Tuberculosis

Spinal manifestations of tuberculosis can present with a myriad of complications, such as tuberculous radiculomyelitis, spinal tuberculoma, myelitis, syringomyelia, vertebral tuberculosis, and rarely spinal tuberculous abscess [39, 40]. The most characteristic spinal complication of tuberculous meningitis is tuberculous arachnoiditis, which can lead to myeloradiculopathy [40].

**Fig. 5:**
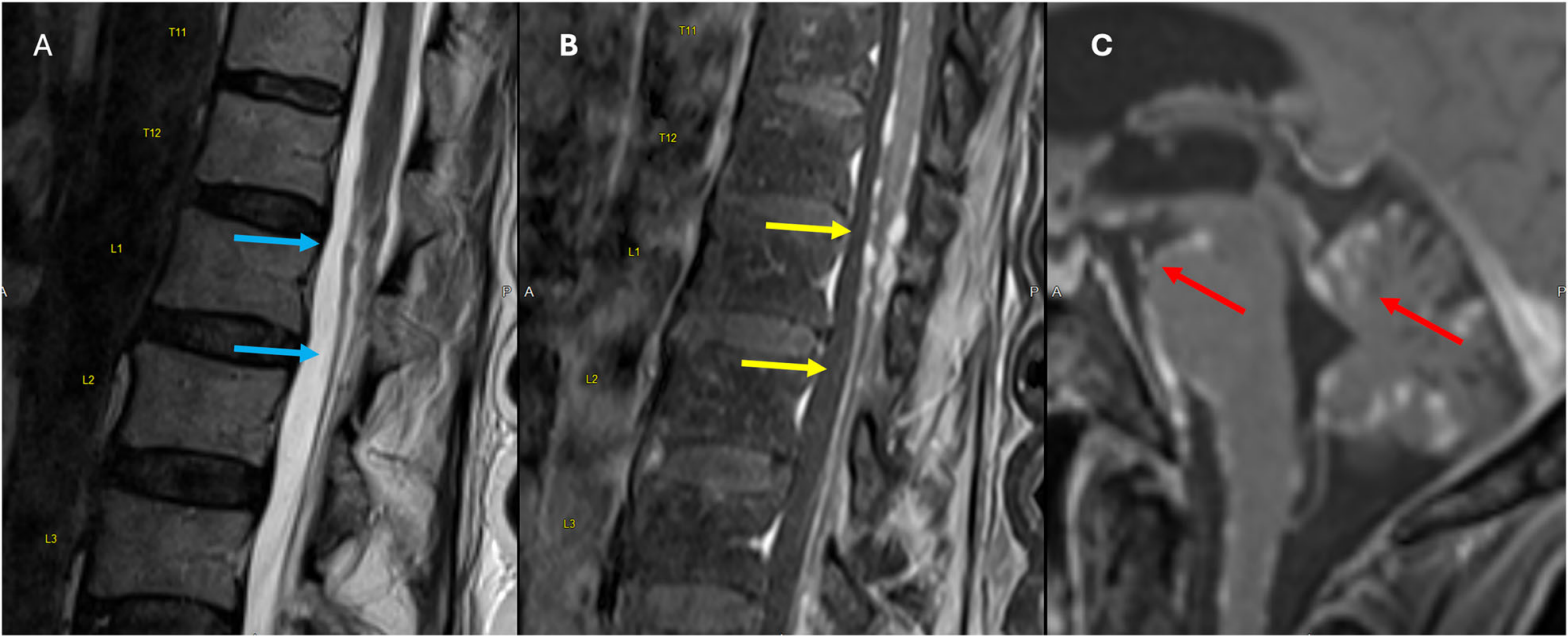
Sixty-six-year-old female presenting with progressive weakness and functional decline at home over the last few months. **A** (Sagittal T2W) and **B** (Sagittal T1 Fatsat postcontrast). Diffuse abnormal nodular T2 hypointensity (blue arrows) and leptomeningeal nodular enhancement (yellow arrows) along the entire surface of the conus and the cauda equina roots. **C** Diffuse leptomeningeal enhancement in the posterior fossa (red arrows)

In patients with spinal tuberculous meningitis, thick exudates present around the distal segment of the spinal cord and conus, as well as around the lumbosacral nerve roots (Fig. 5), cause symptoms similar to those encountered in cauda equina syndrome [39, 40].

#### Bacterial spine infection

Bacterial spine infections are one of the potential causes of back pain [41]. Primary pathogens responsible for spine infection include *Staphylococcus* *aureus*, which accounts for more than 50% of cases, and enteric gram-negative bacilli, such as *Escherichia* *coli* [41, 42].

Infections in the spine can involve three distinct anatomical spaces. These include the disk-endplate complex, which can lead to discitis-osteomyelitis; the facet joints, potentially resulting in septic arthritis; and the epidural space, causing epidural abscess [42, 43].

Post-surgical radiculitis, seen on imaging as postcontrast enhancement of the nerve roots of the cauda equina, is a condition observed at least six months following surgery [41–43]. This enhancement is likely due to the disruption of the blood-nerve barrier during surgery or trauma [42, 43]. If a radicular infection is suspected, MRI postcontrast fat-saturated T1 sequences can depict augmented contrast enhancement of the roots (Fig. S2). The clinical history however, is key to the diagnosis as the enhancement is non-specific [42, 43].

### Beyond the usual

#### Schistosomiasis

Schistosoma mansoni and Schistosoma haematobium are the trematode species that can infect the CNS, with a predominant spinal cord involvement [44–46]. It is important to note that the clinical features of these infections are not specific, and a diagnosis requires epidemiological data and laboratory investigations.

**Fig. 6:**
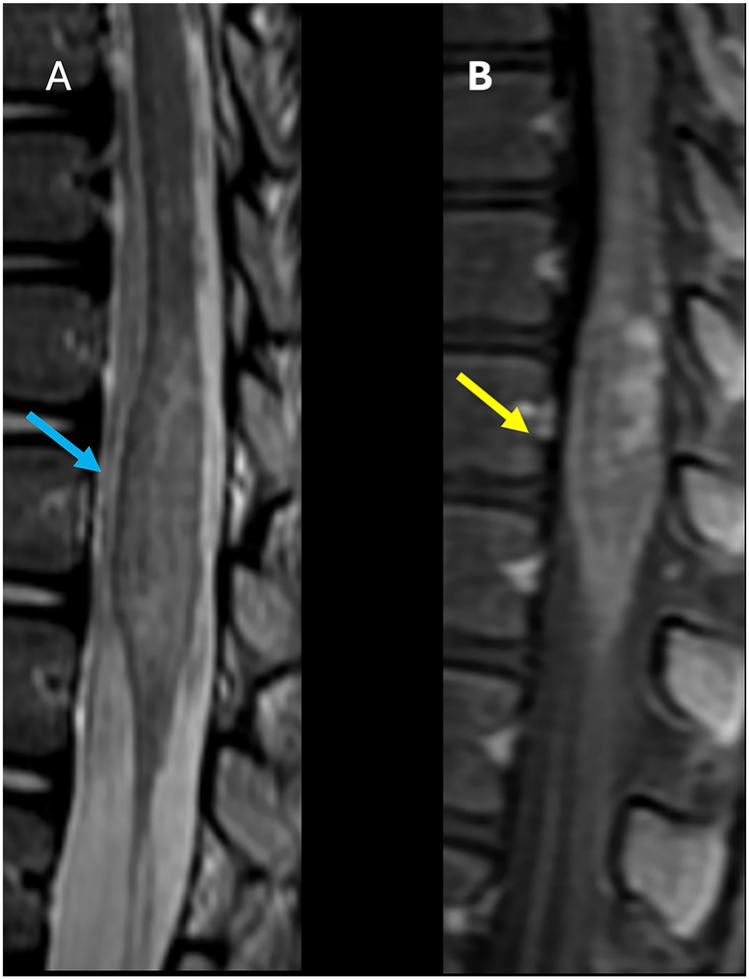
Sixteen-year-old female with bilateral progressive paresthesia in both lower limbs. **A** Sagittal T2W image showing significant expansion and abnormal signal of the conus and abnormal signal on T2. **B** Sagittal T1 postcontrast image showing associated areas of patchy nodular enhancement

On imaging, conus expansion (Fig. 6A), nodular and linear intramedullary enhancement has been described and referred to as “arborized pattern of enhancement” (Fig. 6B) [46]. These changes usually regress with clinical improvement after treatment [45]. Atrophy of the spinal cord may occur in cases of long-term disease [45, 46].

#### Cysticercosis

Neurocysticercosis arises from CNS infection with the pork tapeworm, *Taenia* *solium*, endemic in most low-income countries where pigs are reared. This form of cysticercosis constitutes a significant factor contributing to seizures in endemic regions [44, 47, 48]. Intraspinal neurocysticercosis is a rare condition with a varying incidence of 1–3% [47]. It is usually accompanied by lesions in the brain due to the dissemination of CSF [47, 48]. Isolated spinal cysticercosis is extremely rare [47, 48]. The majority of spinal cysticercosis is extramedullary intradural in nature [48].

Typical MRI features of cysticercosis in the vesicular phase include like in the brain, a cystic lesion with a well-defined thin wall, and a mural nodule that represents the scolex. In this stage, there is no evidence of postcontrast enhancement. Arachnoiditis is rare and is probably caused by an inflammatory reaction due to the rupture of a cyst into the subarachnoid space [48]. Enhancement can be present in the colloidal phase of the disease or in arachnoiditis [48].

The potential differential diagnoses encompass arachnoid cyst, ependymal cyst, neurenteric cyst, sarcoidosis, and neoplasms. Notably, there are no pathognomonic imaging features [49].

#### Lyme

Lyme disease, also known as borreliosis, is caused by the bacteria Borrelia burgdorferi and is transmitted through tick bites [50]. The disease has 3 stages: early localized, early disseminated, and late disseminated [50, 51]. The early stages may present with a rash, flu-like symptoms, and neurologic or cardiac abnormalities. Late dissemination may lead to arthritis and neurologic deficits [50, 51].

Neurologic manifestations include meningoradiculitis, plexus neuritis, CNS involvement, and ocular manifestations [50–53]. Facial nerve involvement is common, and CNS involvement can lead to encephalitis [50, 53]. Ocular manifestations range from conjunctivitis to chronic intraocular inflammation [53].

**Fig. 7:**
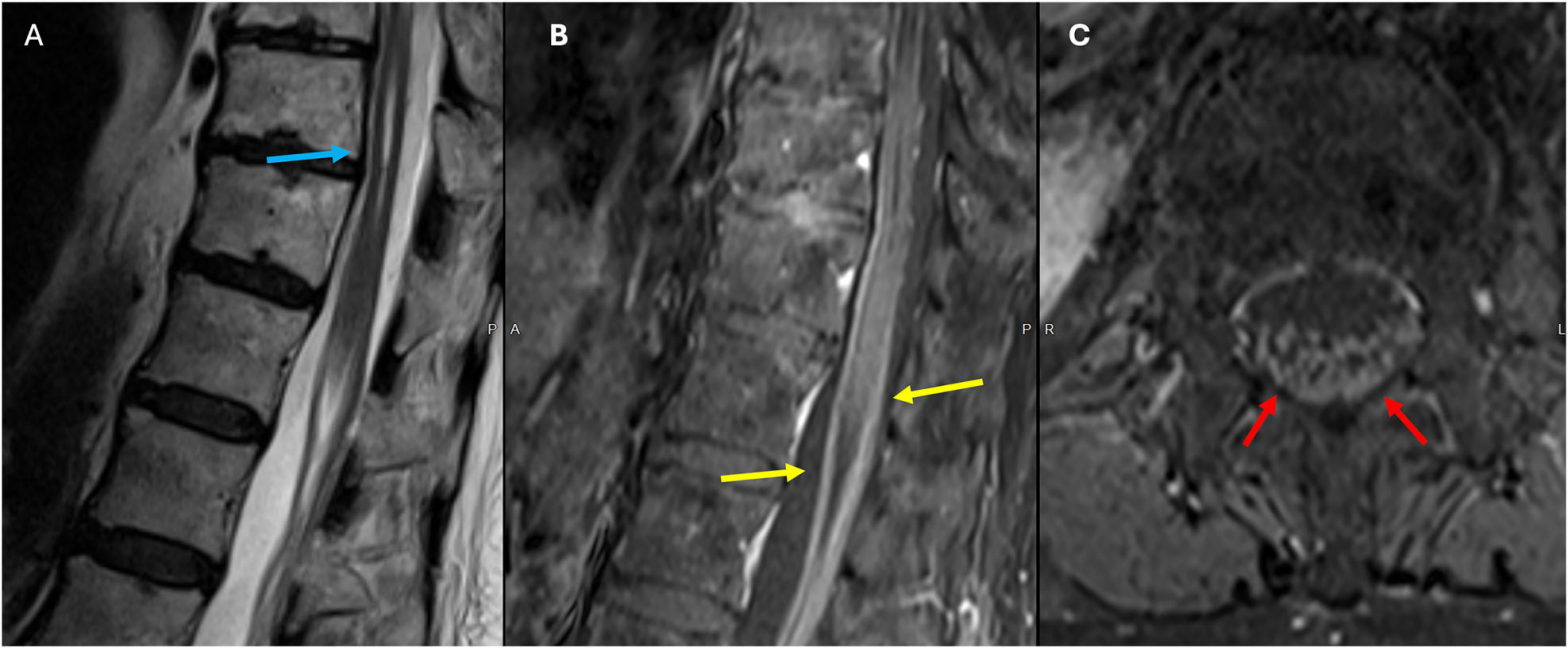
Eighty-three-year-old female who presented with recurrent abdominal pain and hypothyroidism. **A** Syrinx of the central cord extending from T11 to T12 (blue arrow). **B** (sagittal T1 postcontrast) and **C** (axial T1 postcontrast) showing diffuse leptomeningeal enhancement surrounding the spinal cord and the conus (yellow arrows), as well as thickening and enhancement of the cauda equina nerve roots (red arrows)

In Lyme disease, the spinal cord is rarely affected. When it does occur, it is characterized by diffuse or multifocal areas of T2 prolongation and associated nerve root enhancement (Fig. 7) [50, 52].

## Inflammatory

### The usual suspects

#### Guillain-Barré syndrome (GBS)

GBS is a group of autoimmune polyradiculopathies that affect the sensory, motor, and autonomic nerves, causing rapidly progressive flaccid paralysis [54, 55].

GBS is usually preceded by upper respiratory tract infections or diarrhea that occur one to three weeks before the onset of symptoms [55]. Other factors that may increase the likelihood of developing GBS include recent surgery, lymphoma, and systemic lupus erythematosus (SLE) [55].

The classical symptoms of GBS are symmetrical ascending weakness, hyporeflexia, and variable sensory or autonomic involvement [55]. It has different subtypes, with the most common one being acute inflammatory demyelinating polyradiculoneuropathy (AIDP), which is often used interchangeably with GBS [54, 55].

**Fig. 8:**
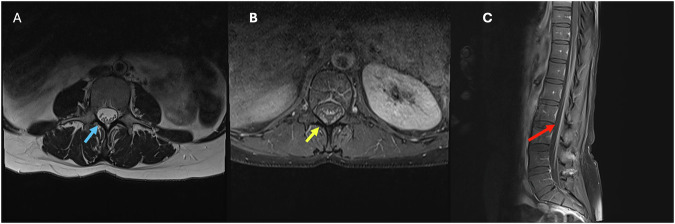
Forty-five-year-old male who presented with symmetrical ascending weakness and areflexia. **A** Axial T2 image of the lumbar spine showing thickening of the cauda equina nerve roots (blue arrow). **B** (axial T1 postcontrast) and **C** (sagittal T1 postcontrast) showing diffuse thickening and enhancement of the nerve roots surrounding the conus medullaris (yellow arrow), as well as thickening and enhancement of the cauda equina nerve roots (red arrow)

Thickening (Fig. 8A) and contrast enhancement of spinal nerve roots (Fig. 8B, C) in the cauda and conus medullaris are typical imaging findings. Although the anterior nerve roots are the most commonly affected site, enhancement of the posterior nerve roots can also be observed [54]. Intracranially, the facial nerve (CN VII) is the most frequently affected cranial nerve [54, 55].

#### Sarcoidosis

Neurosarcoidosis is an uncommon manifestation of sarcoidosis, which can involve the nervous system in various ways, leading to peripheral or cranial neuropathy, or disease. Cauda equina involvement by sarcoidosis is particularly rare [56].

The preferred imaging technique for the initial diagnosis is an MRI of the entire spine with and without contrast [56, 57]. Characteristic findings include nodularity of the spinal nerve roots, diffuse involvement of the cauda equina, and often simultaneous leptomeningeal and pachymeningeal enhancement (Fig. S3) [57].

### Beyond the usual

#### Chronic inflammatory demyelinating polyradiculoneuropathy (CIDP)

CIDP is generally considered a chronic form of GBS that affects peripheral nerves. It causes gradual and prolonged muscle weakness, areflexia, and sensory changes lasting over two months [58, 59]. Whole spine MRI is the imaging modality of choice used to identify CIDP, characterized by thickening and enhancement of the peripheral nerves, brachial and lumbosacral plexus, and nerve roots of the cauda equina [58, 59].

Muscles supplied by the affected nerves show acute to subacute changes in T2 signal and increased enhancement (Fig. S4). Fatty atrophy may occur in chronic cases [58]. Cranial and intercostal nerve involvement is uncommon but possible in some patients [58, 59].

#### Myelin oligodendrocyte glycoprotein antibody-associated disease (MOGAD)

MOGAD is an inflammatory demyelinating disorder characterized by the presence of IgG antibodies to myelin oligodendrocyte (MOG) [60]. Clinical presentation is similar to other demyelinating conditions [60, 61]. Presentations include optic neuritis, transverse myelitis, acute disseminated encephalomyelitis-like encephalomyelitis, cortical encephalitis, and infratentorial syndromes [60–62].

MOGAD usually affects both the gray matter and the central white matter. Lesions can be extensive (longitudinally extensive spinal cord lesions) or involve a short segment [60].

The involvement of both gray and white matter forms the H sign on axial view, and thin linear T2 hyperintense signals on sagittal view [60].

**Fig. 9:**
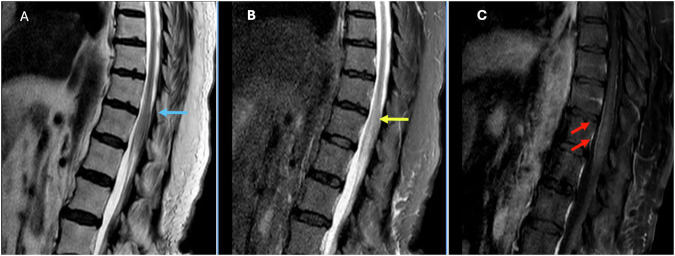
Fifty-two-year-old male who presented with saddle anesthesia and incontinence. **A** Sagittal T2 image of the lumbar spine demonstrates mild expansion within the conus medullaris. (blue arrow). **B** Sagittal STIR showing subtle abnormal high signal intensity (yellow arrow). **C** Sagittal T1 postcontrast showing diffuse “cloud-like” enhancement of the conus medullaris (red arrow)

The lower cord is often preferentially affected, with the conus medullaris being classically affected (Fig. 9A, B) [60, 62]. The presence of linear T2-hyperintensity in the central canal is a commonly observed transient radiologic feature in MOGAD and neuromyelitis optica spectrum disorder (NMOSD) but not in Multiple Sclerosis (MS) [63].

A helpful distinguishing feature is the presence of leptomeningeal enhancement, relatively common in MOGAD is rarely seen in MS and NMOSD. Additionally, contrast-enhanced sequences may reveal heterogeneous enhancement with blurred margins, referred to as “cloudlike” enhancement (Fig. 9C), as well as a pattern of linear, ‘pencil-thin’ enhancement of the ependyma [60, 62].

## Vascular

### The usual suspects

#### Spinal dural arteriovenous fistulas

Spinal dural arteriovenous fistulas (SDAVFs) are the most common type of spinal vascular malformation [64, 65]. Diagnosis is challenging, and symptoms develop over months to years and include pain, weakness, sensory changes, and sphincter dysfunction, known as Foix–Alajouanine syndrome [66].

**Fig. 10:**
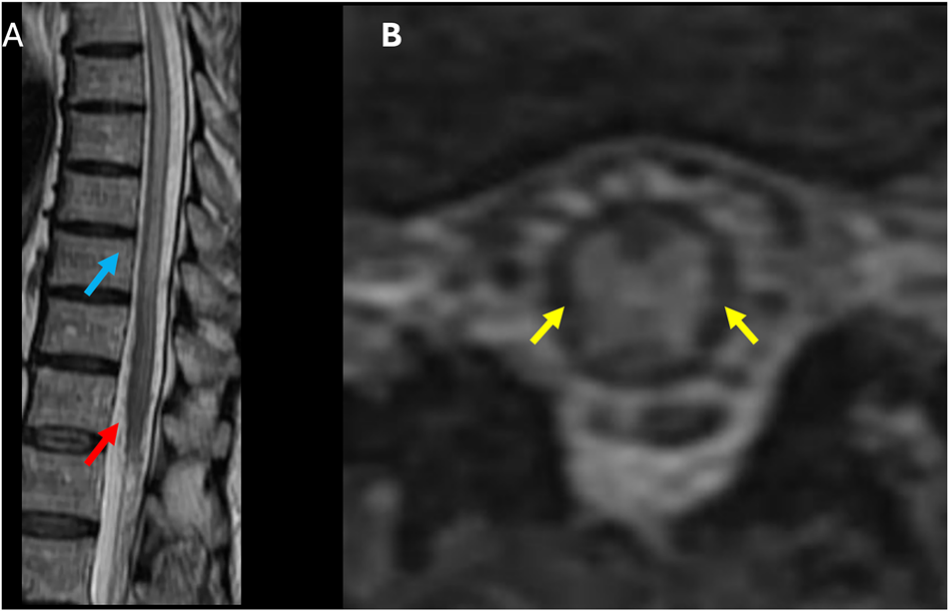
Eighty-eight-year-old male who presented with progressive pain, lower extremity weakness, or sensory changes. **A** Sagittal T2 image of the thoracolumbar spine showing mild expansion and longitudinally extensive high signal intensity within the distal cord and conus medullaris (blue arrow), as well as prominent serpiginous intradural extramedullary flow voids (red arrow). **B** Axial T2 image of the lumbar spine demonstrates expansion and centrally located high signal intensity within the conus medullaris (yellow arrows)

Diffuse multilevel intramedullary hyperintensity occurs due to edema [64, 66]. Regardless of the fistula’s location, the T2 hyperintensity involves the conus medullaris in up to 90% of cases, primarily due to orthostasis (Fig. 10A) [65]. However, the segmental level of cord enlargement and signal change does not necessarily correlate with the location of the fistula [64, 67].

The most specific finding is the presence of prominent serpiginous intradural extramedullary flow voids along the dorsal aspect of the cord (Fig. 10B), which, if large enough, may cause the cord’s surface to appear scalloped [68, 69]. Patchy intramedullary enhancement could be seen, as well as serpentine enhancement of the perimedullary veins along the cord surface [64–69].

The gold standard for the final diagnosis of spinal dural arteriovenous fistulas is catheter-based spinal angiography (digital subtraction angiography). This technique allows precise identification of the fistula’s location, feeding arteries, and venous drainage pattern, thereby guiding appropriate treatment [64, 66, 70].

CT angiography is increasingly utilized and has shown high sensitivity in localizing the fistula, especially with advanced multi-detector and 3D techniques. Recent literature and imaging guidelines acknowledge that CT angiography can successfully localize SDAVF in up to 75–78% of cases, making it an important tool, especially when conventional spinal angiography is technically challenging or contraindicated [71].

#### Infarct

Spinal cord ischemia is a rare condition with a poor prognosis [72] that can lead to sudden and severe paraplegia and paraparesis, with quadriplegia or tetraplegia observed in higher cord lesions [72, 73]. Most patients experience sensory disturbance, and urinary catheterization is required in most patients [73].

The supply of the conus medullaris primarily depends on the artery of Adamkiewicz, which in approximately 76% originates from the left side of the aorta, between the T8 and L1 segments, and connects with the anterior spinal artery [72, 74].

**Fig. 11:**
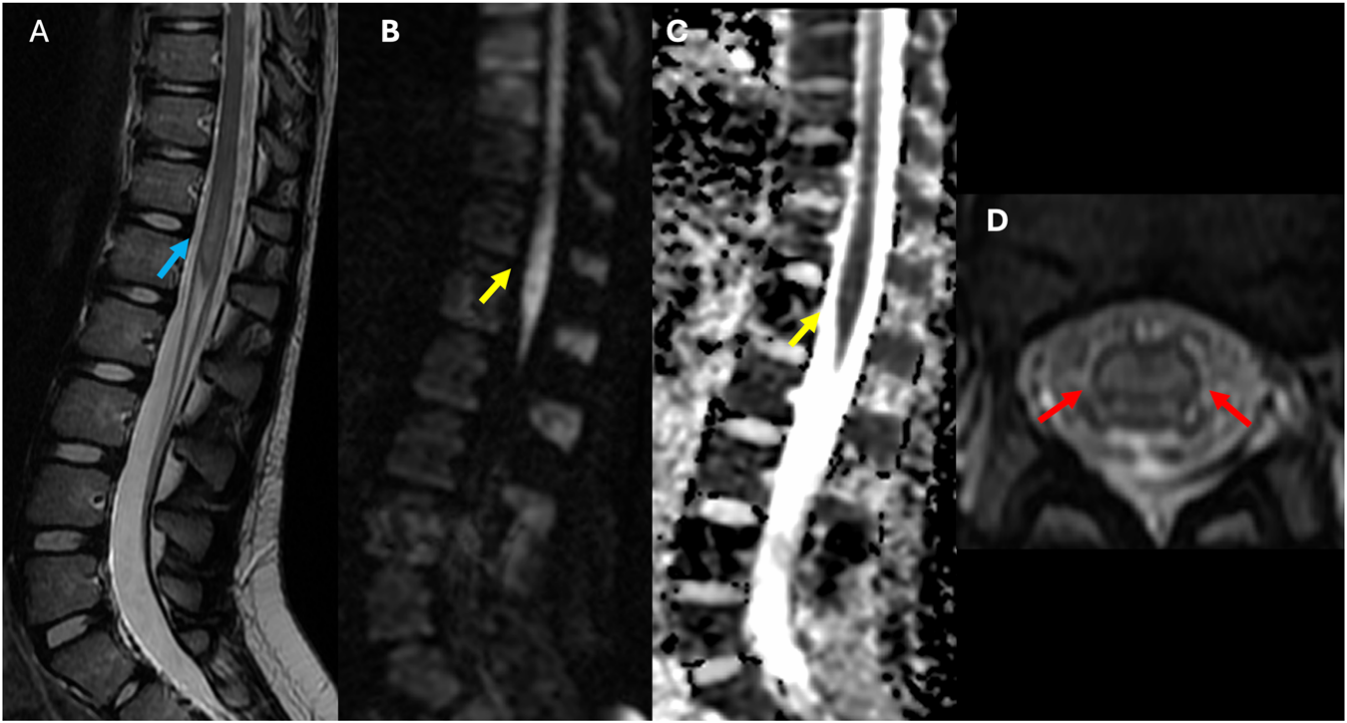
Forty-one-year-old male who presented with acute lower extremity weakness and back pain. **A** Sagittal T2 image of the lumbar spine demonstrates mild expansion and diffuse abnormal high signal intensity within the distal cord and conus medullaris. (blue arrow). **B** (DWI) and **C** (ADC) showing diffusion restriction in the conus medullaris (yellow arrow). **D** Axial T2 image of the lumbar spine demonstrates expansion and centrally located high signal intensity within the conus medullaris (red arrow)

An abnormal T2 signal within the cord is the primary indicator of spinal cord infarction, and the signal pattern will vary depending on the affected territory (Fig. 11A, D) [72, 75].

- Anterior spinal artery infarct: typically manifests as bilateral T2 hyperintensity involving the anterior two-thirds of the cord, most evident on sagittal and axial images with the classic “owl’s eye” sign on axial T2. Clinical signs are often severe motor deficits with or without dissociated sensory loss [8, 75, 76].
- Posterior spinal artery infarct: T2 hyperintensity localized to the dorsal aspect of the cord, presenting with proprioceptive loss and sensory ataxia, but relative preservation of motor function [72].
- Central infarct: involvement of the central cord, sometimes secondary to venous infarction or systemic hypoperfusion, can show symmetric T2 hyperintensity with or without cord expansion [72, 76].

Diffusion-weighted imaging is increasingly used and can reveal restricted diffusion (Fig. 11B, C) [75]. Vertebral body infarction is present in 9-30% of the cases and can be a useful confirmatory sign of spinal cord infarct [77].

### Beyond the usual

#### Arteriovenous malformation (AVM)

Spinal AVMs are rare. They are characterized by an abnormal connection between arteries and veins, with an intervening nidus [78]. They have a clinical presentation that is similar to spinal dural arteriovenous fistulas (SDAVFs) [70, 78].

Angiography is the preferred imaging method of investigation, but requires careful technique [70]. It is important to note that the arterial supply may arise anywhere from the upper thoracic region to the sacral areas if the lesion is around the conus, with little correlation to the clinical level or visible nidus [70, 78].

The T1W sequences signal voids are observed due to high-velocity flow, as well as cord indentation/scalloping caused by the dilated perimedullary vessels [69, 70, 78]. The T2 signal voids are also due to high-velocity flow. The T2 hyperintensity in the cord signal can result from edema or myelomalacia [70, 78].

## Miscellanea

### Ventriculus terminalis

The ventriculus terminalis is a fusiform dilation of the spinal cord’s terminal central canal, located at the tip of the conus medullaris and lined with ependyma [79]. It should not be confused with a filar cyst [79, 80]. Regardless of the imaging modality used to visualize the spine, a ventriculus terminalis in newborns presents as a cystic structure at the tip of the conus medullaris, typically extending over 8–10 mm with a transverse diameter of 2–4 mm. In childhood, it often persists as a small cystic structure but is seldom identifiable in adults.

The MRI features typically include fluid signal characteristics. On T1W images, it usually appears hypointense, while on T2W images, it typically appears hyperintense (Fig. S5). Additionally, on T1 postcontrast images, it generally does not show enhancement.

### Diastematomyelia

Diastematomyelia is a rare type of spinal dysraphism (spina bifida occulta) that occurs when the spinal cord has a longitudinal split [81, 82]. The clinical manifestations are nonspecific and include cutaneous abnormalities overlying the spine, neurologic deficits, and orthopedic abnormalities [83].

**Fig. 12:**
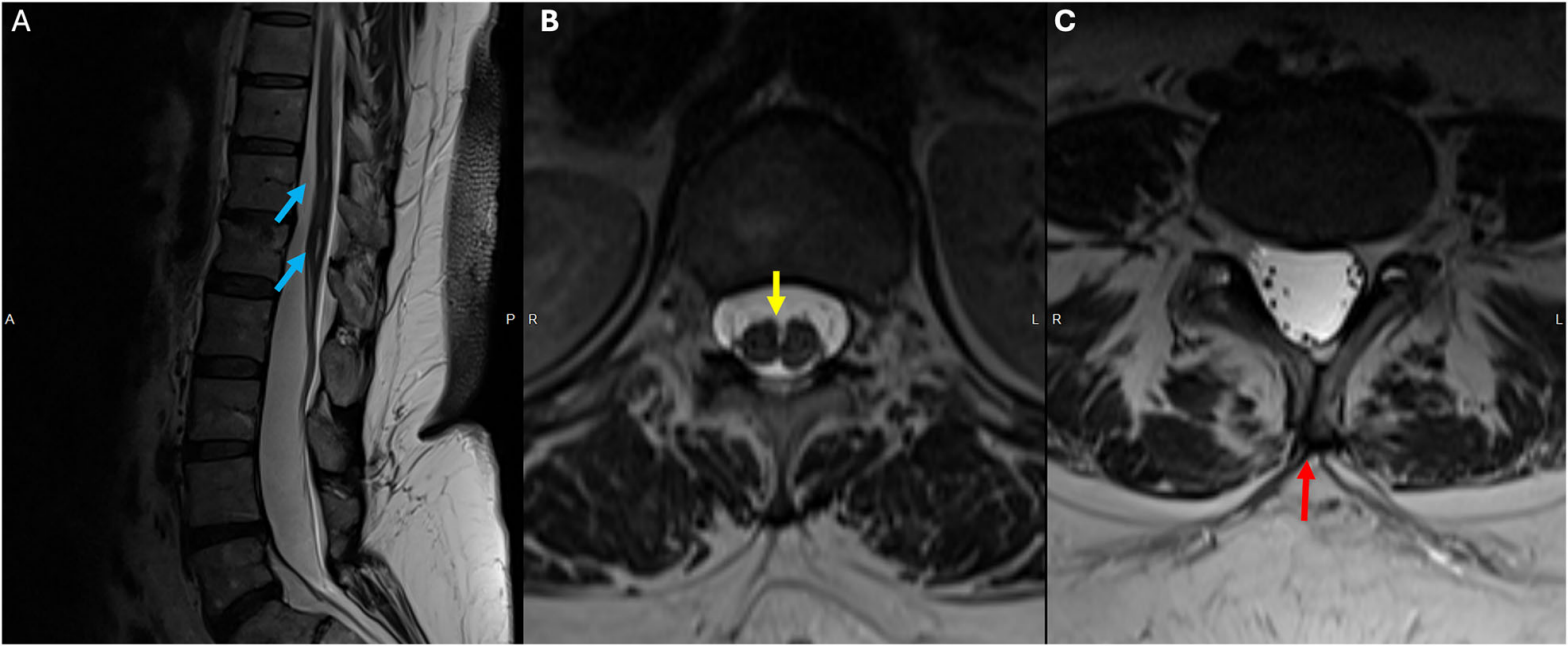
Forty-seven-year-old female who presented with back pain. **A** Sagittal T2 showing focal central syrinx at the T12 and the L1 level (blue arrows). **B** Duplicated dural sac separated by midline spur (yellow arrow). **C** Axial T2 showing incomplete fusion of the posterior elements of L5 (red arrow)

Split cord malformations are divided into two types based on the dural sac division [82, 83]. Type I features a duplicated dural sac with a midline rigid osseous or cartilaginous spur that is usually symptomatic (Fig. 12) [82]. Type II has a single dural sac with a midline nonrigid fibrous or fibrovascular septum, and impairment is less marked [82].

A third of cases have an associated bony, cartilaginous, or fibrous spur projecting through the dura mater forwards from the neural arch. Vertebral anomalies like spina bifida, butterfly, or hemivertebrae are common [81–83].

During an antenatal ultrasound, the identification of an additional echogenic focus in the midline between the fetal spinal posterior elements has been established as a reliable indicator.

Recent literature and case reports confirm that diastematomyelia can present de novo or be incidentally discovered in adulthood, sometimes as isolated findings or with non-specific neurological symptoms or tethered cord syndrome [84].

MRI is the preferred imaging modality for evaluating split cord malformations. It can accurately depict the spinal cord and detect the presence of hydromyelia, if present. Additionally, it can assess for the presence of various associated anomalies, such as a meningocele or a dermoid cyst.

### Degenerative spine disease

The contrast enhancement of MR imaging depends on three factors: the intravascular component, the extravascular component, and the relaxation time properties of the tissues [85].

The capillaries of the cauda equina have a blood-nerve barrier, which prevents Gd-DTPA from leaking out of the vessels in the normal state [85, 86]. However, if there is cauda equina compression due to canal stenosis, intraradicular circulatory disturbance and nerve degeneration can lead to breakdown of the blood-nerve barrier [85, 86]. This breakdown can cause intraradicular edema, which can lead to subsequent enhancement on MRI images postcontrast (Fig. S6) [86].

## Conclusion

Patients with a wide variety of benign and malignant conditions, may present with conus or cauda equina symptoms, including perianal and “saddle” paresthesia. bowel, bladder, and/or sexual dysfunction, low back pain, radiculopathy, paresthesia or weakness of the lower limbs, and abnormal lower limb reflexes. Contrast-enhanced MRI of the spine is the imaging modality of choice for the detection and evaluation of the underlying cause. Radiologists should be familiar with the imaging features of common and infrequent pathologies that may affect the conus and the cauda equina to help narrow the differential diagnosis and guide treatment.

## Supplementary information

ELECTRONIC SUPPLEMENTARY MATERIAL

## Abbreviations

CIDP: Chronic inflammatory demyelinating polyradiculoneuropathy
CNS: Central nervous system
CSF: Cerebrospinal fluid
GBM: Glioblastoma multiforme
GBS: Guillain–Barré syndrome
LM: Leptomeningeal metastases
MOGAD: Myelin oligodendrocyte glycoprotein antibody-associated disease
MRI: Magnetic resonance imaging
MS: Multiple sclerosis
NMOSD: Neuromyelitis optica spectrum disorder
STIR: Short tau inversion recovery
T1W: T1-weighted (MRI sequence)
T2W: T2-weighted (MRI sequence)
WHO: World Health Organization

## References

[1] Vaishya S, Pojskic M, Bedi MS et al (2024) Cauda equina, conus medullaris and syndromes mimicking sciatic pain: WFNS spine committee recommendations. World Neurosurg X 22:100274. 10.1016/j.wnsx.2024.100274 10.1016/j.wnsx.2024.100274 PMC10943478 38496349

[2] Harrop JS, Hunt GE, Vaccaro AR (2004) Conus medullaris and cauda equina syndrome as a result of traumatic injuries: management principles. Neurosurg Focus 16:1–23. 10.3171/FOC.2004.16.6.4 10.3171/foc.2004.16.6.4 15202874

[3] Gitelman A, Hishmeh S, Morelli BN et al (2008) Cauda equina syndrome: a comprehensive review. Am J Orthop 37:556–562 19104682

[4] McNamee J, Flynn P, O’Leary S, Love M, Kelly B (2013) Imaging in cauda equina syndrome—a pictorial review. Ulst Med J 82:100 PMC3756868 24082289

[5] Kunam VK, Velayudhan V, Chaudhry ZA, Bobinski M, Smoker WRK, Reede DL (2018) Incomplete cord syndromes: clinical and imaging review. Radiographics 38:1201–1222. 10.1148/RG.2018170178 10.1148/rg.2018170178 29995620

[6] Rounds G, Mcnamee J, Flynn P, Love M, Kelly B (2013) Imaging in cauda equina syndrome—a pictorial review. Ulster Med J 82:100–108 PMC3756868 24082289

[7] Rider LS, Marra EM (2025) Cauda equina and conus medullaris syndromes. StatPearls Publishing, Treasure Island 30725885

[8] Da Ros V, Picchi E, Ferrazzoli V et al (2021) Spinal vascular lesions: anatomy, imaging techniques and treatment. Eur J Radiol Open. 10.1016/j.ejro.2021.100369 10.1016/j.ejro.2021.100369 PMC8283341 34307789

[9] Soderlund KA, Smith AB, Rushing EJ, Smirniotopolous JG (2012) Radiologic-pathologic correlation of pediatric and adolescent spinal neoplasms: part 2, intradural extramedullary spinal neoplasms. AJR Am J Roentgenol 198:44–51. 10.2214/AJR.11.7121 22194478 10.2214/AJR.11.7121

[10] Abul-Kasim K, Thurnher MM, McKeever P, Sundgren PC (2008) Intradural spinal tumors: current classification and MRI features. Neuroradiology 50:301–314. 10.1007/S00234-007-0345-7 18084751 10.1007/s00234-007-0345-7

[11] Louis DN, Perry A, Wesseling P et al (2021) The 2021 WHO classification of tumors of the central nervous system: a summary. Neuro Oncol 23:1231–1251. 10.1093/NEUONC/NOAB106 34185076 10.1093/neuonc/noab106 PMC8328013

[12] Koeller KK, Shih RY (2019) Intradural extramedullary spinal neoplasms: radiologic-pathologic correlation. Radiographics 39:468–490. 10.1148/RG.2019180200 30844353 10.1148/rg.2019180200

[13] Schellinger KA, Propp JM, Villano JL, McCarthy BJ (2008) Descriptive epidemiology of primary spinal cord tumors. J Neurooncol 87:173–179. 10.1007/S11060-007-9507-Z 18084720 10.1007/s11060-007-9507-z

[14] Weber DC, Wang Y, Miller R et al (2014) Long-term outcome of patients with spinal myxopapillary ependymoma: treatment results from the MD Anderson Cancer Center and institutions from the rare cancer network. Neuro Oncol 17:588–595. 10.1093/NEUONC/NOU293 25301811 10.1093/neuonc/nou293 PMC4483075

[15] Lenzi J, Anichini G, Landi A et al (2017) Spinal nerves schwannomas: experience on 367 cases—historic overview on how clinical, radiological, and surgical practices have changed over a course of 60 years. Neurol Res Int. 10.1155/2017/3568359 10.1155/2017/3568359 PMC5624174 29075532

[16] Demachi H, Takashima T, Kadoya M et al (1990) MR imaging of spinal neurinomas with pathological correlation. J Comput Assist Tomogr 14:250–254. 10.1097/00004728-199003000-00017 2312854 10.1097/00004728-199003000-00017

[17] Gu R, Liu JB, Zhang Q, Liu GY, Zhu QS (2014) MRI diagnosis of intradural extramedullary tumors. J Cancer Res Ther 10:927–931. 10.4103/0973-1482.137993 25579530 10.4103/0973-1482.137993

[18] Lee JH, Kim HS, Yoon YC, Cha MJ, Lee SH, Kim ES (2020) Differentiating between spinal schwannomas and meningiomas using MRI: a focus on cystic change. PLoS One. 10.1371/JOURNAL.PONE.0233623 10.1371/journal.pone.0233623 PMC7259580 32469953

[19] Zhai X, Zhou M, Chen H et al (2019) Differentiation between intraspinal schwannoma and meningioma by MR characteristics and clinic features. Radiol Med 124:510–521. 10.1007/S11547-019-00988-Z 30684254 10.1007/s11547-019-00988-z

[20] Hirano K, Imagama S, Sato K et al (2012) Primary spinal cord tumors: review of 678 surgically treated patients in Japan. A multicenter study. Eur Spine J 21:2019–2026. 10.1007/S00586-012-2345-5 22581192 10.1007/s00586-012-2345-5 PMC3463691

[21] Wang N, Bertalan MS, Brastianos PK (2018) Leptomeningeal metastasis from systemic cancer: review and update on management. Cancer 124:21–35. 10.1002/cncr.30911 29165794 10.1002/cncr.30911 PMC7418844

[22] Le Rhun E, Galanis E (2016) Leptomeningeal metastases of solid cancer. Curr Opin Neurol 29:797–805. 10.1097/WCO.0000000000000393 27661208 10.1097/WCO.0000000000000393

[23] Passarin MG, Sava T, Furlanetto J et al (2015) Leptomeningeal metastasis from solid tumors: a diagnostic and therapeutic challenge. Neurol Sci 36:117–123. 10.1007/s10072-014-1881-7 25022241 10.1007/s10072-014-1881-7

[24] Kaya S, Lam FC, Stevenson MA, Motiei-Langroudi R, Kasper EM (2024) Intramedullary metastases to conus medullaris: a review of the literature with a case illustration. Brain Sci. 10.3390/brainsci14080761 10.3390/brainsci14080761 PMC11352703 39199455

[25] Nguyen A, Nguyen A, Dada OT et al (2023) Leptomeningeal metastasis: a review of the pathophysiology, diagnostic methodology, and therapeutic landscape. Curr Oncol 30:5906–5931. 10.3390/curroncol30060442 37366925 10.3390/curroncol30060442 PMC10297027

[26] Wright CH, Wright J, Onyewadume L et al (2019) Diagnosis, treatment, and survival in spinal dissemination of primary intracranial glioblastoma: systematic literature review. J Neurosurg Spine 31:723–732. 10.3171/2019.5.SPINE19164 31374545 10.3171/2019.5.SPINE19164

[27] Lawton CD, Nagasawa DT, Yang I, Fessler RG, Smith ZA (2012) Leptomeningeal spinal metastases from glioblastoma multiforme:Treatment and management of an uncommon manifestation of disease: a review. J Neurosurg Spine 17:438–448. 10.3171/2012.7.SPINE12212 22958073 10.3171/2012.7.SPINE12212

[28] Solomou AG (2017) Magnetic resonance imaging of pineal tumors and drop metastases: a review approach. Rare Tumors 9:69–76. 10.4081/RT.2017.6715 10.4081/rt.2017.6715 PMC5661140 29142658

[29] Dumrongpisutikul N, Intrapiromkul J, Yousem DM (2012) Fellows’ journal club: distinguishing between germinomas and pineal cell tumors on MR imaging. AJNR Am J Neuroradiol 33:550. 10.3174/AJNR.A2806 22173760 10.3174/ajnr.A2806 PMC7966430

[30] Ryu SM, Lee SH, Kim ES, Eoh W (2018) Predicting survival of patients with spinal ependymoma using machine learning algorithms with the SEER database. World Neurosurg 124:e331–e339. 10.1016/J.WNEU.2018.12.091 30597279 10.1016/j.wneu.2018.12.091

[31] Armstrong TS, Vera-Bolanos E, Bekele BN, Aldape K, Gilbert MR (2010) Adult ependymal tumors: prognosis and the M. D. Anderson Cancer Center experience. Neuro Oncol 12:862–870. 10.1093/NEUONC/NOQ009 20511182 10.1093/neuonc/noq009 PMC2940672

[32] Villanueva-Castro E, Meraz-Soto JM, Hernández-Dehesa IA et al (2023) Spinal ependymomas: an updated WHO classification and a narrative review. Cureus. 10.7759/CUREUS.49086 10.7759/cureus.49086 PMC10731541 38125233

[33] Hsu KC, Li TY, Chu HY, Chen LC, Chang ST, Wu YT (2013) Conus medullaris metastasis in breast cancer: report of a case and a review of the literature. Surg Today 43:910–914. 10.1007/S00595-012-0289-3 22872491 10.1007/s00595-012-0289-3

[34] Mavani S, Nadkarni T, Goel N (2013) Intramedullary conus metastasis from carcinoma lung. J Craniovertebr Junction Spine 4:40–42. 10.4103/0974-8237.121626 24381457 10.4103/0974-8237.121626 PMC3872662

[35] Akyildiz A, Yildirim HC, Ismayilov R, Isikay AI, Kertmen N (2023) Conus medullaris metastasis in a patient with triple-positive breast cancer. Cauc Med J 1:39–41. 10.4274/cmj.galenos.2023.53825

[36] Wu Z, Xu S, Zhong C et al (2014) Intramedullary conus medullaris metastasis from prostate carcinoma: a case report and review of the literature. Oncol Lett 7:717. 10.3892/OL.2014.1808 24527078 10.3892/ol.2014.1808 PMC3919891

[37] Sanborn MR, Pramick M, Brooks J, Welch WC (2011) Glioblastoma multiforme in the adult conus medullaris. J Clin Neurosci 18:842–843. 10.1016/J.JOCN.2010.08.037 21435883 10.1016/j.jocn.2010.08.037

[38] Shen CX, Wu JF, Zhao W, Cai ZW, Cai RZ, Chen CM (2017) Primary spinal glioblastoma multiforme: A case report and review of the literature. Medicine (Baltimore) 10.1097/MD.0000000000006634 10.1097/MD.0000000000006634 PMC5406076 28422860

[39] Hernández-Albújar S, Arribas JR, Royo A, González-García JJ, Peña JM, Vázquez JJ (2000) Tuberculous radiculomyelitis complicating tuberculous meningitis: case report and review. Clin Infect Dis 30:915–921. 10.1086/313821 10854362 10.1086/313821

[40] Garg RK, Malhotra HS, Gupta R (2015) Spinal cord involvement in tuberculous meningitis. Spinal Cord 53:649–657. 10.1038/sc.2015.58 25896347 10.1038/sc.2015.58

[41] Laur O, Mandell JC, Titelbaum DS, Cho C, Smith SE, Khurana B (2019) Acute nontraumatic back pain: infections and mimics. Radiographics 39:287–288. 10.1148/RG.2019180077 30620695 10.1148/rg.2019180077

[42] Corona-Cedillo R, Saavedra-Navarrete MT, Espinoza-Garcia JJ, Mendoza-Aguilar AN, Ternovoy SK, Roldan-Valadez E (2021) Imaging assessment of the postoperative spine: an updated pictorial review of selected complications. Biomed Res Int 2021:9940001. 10.1155/2021/9940001 34113681 10.1155/2021/9940001 PMC8154286

[43] Ledbetter LN, Salzman KL, Shah LM, Ledbetter XLN, Salzman XKL, Shah XLM (2016) Imaging psoas sign in lumbar spinal infections: evaluation of diagnostic accuracy and comparison with established imaging characteristics. AJNR Am J Neuroradiol 37:736–741. 10.3174/AJNR.A4571 26585257 10.3174/ajnr.A4571 PMC7960153

[44] Shih R, Koeller KK (2015) Bacterial, fungal, and parasitic infections of the central nervous system: radiologic-pathologic correlation and historical perspectives: from the radiologic pathology archives. Radiographics 35:1141–1169. 10.1148/RG.2015140317 10.1148/rg.2015140317 26065933

[45] Saleem S, Belal AI, El-Ghandour NM (2005) Spinal cord schistosomiasis: MR imaging appearance with surgical and pathologic correlation. AJNR Am J Neuroradiol 26:1646 16091508 PMC7975157

[46] Ashour A, Elserry T, Nosser M et al (2018) Spinal schistosomiasis: cases in Egyptian population. J Craniovertebr Junction Spine 9:76–80. 10.4103/JCVJS.JCVJS_2_18 29755242 10.4103/jcvjs.JCVJS_2_18 PMC5934970

[47] Hung ND, Duc NM, Sy TV, Dung LT, Tuan TA, Hue ND (2020) Distinct forms of spinal cysticercosis: a vietnamese case series. Curr Med Imaging 17:648–652. 10.2174/1573405616666201118142317 10.2174/1573405616666201118142317 33213334

[48] Zhao JL, Lerner A, Shu Z, Gao XJ, Zee CS (2015) Imaging spectrum of neurocysticercosis. Radiol Infect Dis 1:94–102. 10.1016/J.JRID.2014.12.001

[49] Rajbhandari S, Gurung P, Yadav J, Rajbhandari P, Acharya S, Pant B (2021) A case report of multiple isolated intradural neurocysticercosis of the lumbo-sacral spine. Int J Surg Case Rep 87:106434. 10.1016/J.IJSCR.2021.106434 34562721 10.1016/j.ijscr.2021.106434 PMC8473764

[50] Valand HA, Goyal A, Melendez DA, Matharu SS, Mangat HS, Tu RK (2019) Lyme disease: what the neuroradiologist needs to know. AJNR Am J Neuroradiol. 10.3174/ajnr.A6301 10.3174/ajnr.A6301 PMC6975358 31672835

[51] Shapiro ED (2014) Lyme disease. N Engl J Med 371:683–684. 10.1056/NEJMc1407264 10.1056/NEJMc1407264 PMC4492124 25119621

[52] Agosta F, Rocca MA, Benedetti B, Capra R, Cordioli C, Filippi M (2006) MR imaging assessment of brain and cervical cord damage in patients with neuroborreliosis. AJNR Am J Neuroradiol 27:892 16611786 PMC8133998

[53] Hildenbrand P, Craven DE, Jones R, Nemeskal P (2009) Lyme neuroborreliosis: manifestations of a rapidly emerging zoonosis. AJNR Am J Neuroradiol 30:1079–1087. 10.3174/ajnr.A1579 19346313 10.3174/ajnr.A1579 PMC7051319

[54] Alkan O, Yildirim T, Tokmak N, Tan M (2009) Spinal MRI findings of Guillain–Barré syndrome. J Radiol Case Rep 3:25–28. 10.3941/JRCR.V3I3.153 22470650 10.3941/jrcr.v3i3.153 PMC3303301

[55] Hughes RAC, Cornblath DR (2005) Guillain–Barré syndrome. Lancet 366:1653–1666. 10.1016/S0140-6736(05)67665-9 16271648 10.1016/S0140-6736(05)67665-9

[56] Kaiboriboon K, Olsen TJ, Hayat GR (2005) Cauda equina and conus medullaris syndrome in sarcoidosis. Neurologist 11:179–183. 15860141 10.1097/01.nrl.0000159983.19068.21

[57] Bou GA, Garcia-Santibanez R, Castilho AJ, Hutto SK (2022) Neurosarcoidosis of the cauda equina: clinical course, radiographic and electrodiagnostic findings, response to treatment, and outcomes. Neurol Neuroimmunol Neuroinflamm 9:e1170 10.1212/NXI.0000000000001170 PMC9128042 35487693

[58] Thawait SK, Chaudhry V, Thawait GK et al (2011) High-resolution MR neurography of diffuse peripheral nerve lesions. AJNR Am J Neuroradiol 32:1365–1372. 10.3174/AJNR.A2257 20966057 10.3174/ajnr.A2257 PMC7964353

[59] Schulze M, Kötter I, Ernemann U et al (2009) MRI findings in inflammatory muscle diseases and their noninflammatory mimics. AJR Am J Roentgenol 192:1708–1716. 10.2214/AJR.08.1764/ASSET/IMAGES/06_08_1764_28.JPEG 19457839 10.2214/AJR.08.1764

[60] Shahriari M, Sotirchos ES, Newsome SD, Yousem DM (2021) MOGAD: how it differs from and resembles other neuroinflammatory disorders. AJR Am J Roentgenol 216:1031–1039. 10.2214/AJR.20.24061/ASSET/IMAGES/LARGE/04_20_24061_03B.JPEG 32755221 10.2214/AJR.20.24061

[61] Bartels F, Lu A, Oertel FC, Finke C, Paul F, Chien C (2021) Clinical and neuroimaging findings in MOGAD–MRI and OCT. Clin Exp Immunol 206:266–281. 10.1111/CEI.13641 34152000 10.1111/cei.13641 PMC8561692

[62] dos Passos GR, Oliveira LM, da Costa BK et al (2018) MOG-IgG-associated optic neuritis, encephalitis, and myelitis: Lessons learned from neuromyelitis optica spectrum disorder. Front Neurol 9:323689. 10.3389/FNEUR.2018.00217/BIBTEX 10.3389/fneur.2018.00217 PMC5893792 29670575

[63] Webb LM, Cacciaguerra L, Krecke KN et al (2023) Marked central canal T2-hyperintensity in MOGAD myelitis and comparison to NMOSD and MS. J Neurol Sci 450:120687. 10.1016/J.JNS.2023.120687 37201267 10.1016/j.jns.2023.120687 PMC10492002

[64] Jeng Y, Chen DYT, Hsu HL, Huang YL, Chen CJ, Tseng YC (2015) Spinal dural arteriovenous fistula: imaging features and its mimics. Korean J Radiol 16:1119–1131. 10.3348/KJR.2015.16.5.1119 26357504 10.3348/kjr.2015.16.5.1119 PMC4559784

[65] Huffmann BC, Gilsbach JM, Thron A (1995) Spinal dural arteriovenous fistulas: a plea for neurosurgical treatment. Acta Neurochir 135:44–51. 10.1007/BF02307413 8748791 10.1007/BF02307413

[66] Krings T, Geibprasert S (2009) Spinal dural arteriovenous fistulas. AJNR Am J Neuroradiol 30:639–648. 10.3174/AJNR.A1485 19213818 10.3174/ajnr.A1485 PMC7051782

[67] Jellema K, Tijssen CC, van Gijn J (2006) Spinal dural arteriovenous fistulas: a congestive myelopathy that initially mimics a peripheral nerve disorder. Brain 129:3150–3164. 10.1093/BRAIN/AWL220 16921175 10.1093/brain/awl220

[68] Morris JM (2012) Imaging of dural arteriovenous fistula. Radiol Clin North Am 50:823–839. 10.1016/J.RCL.2012.04.011 22643397 10.1016/j.rcl.2012.04.011

[69] Gilbertson JR, Miller GM, Goldman MS, Marsh WR Spinal dural arteriovenous fistulas: MR and myelographic findings. AJNR Am J Neuroradiol 16:2049–2057 PMC8337217 8585493

[70] Patsalides A, Knopman J, Santillan A, Tsiouris AJ, Riina H, Gobin YP (2011) Endovascular treatment of spinal arteriovenous lesions: beyond the dural fistula. AJNR Am J Neuroradiol 32:798–808. 10.3174/AJNR.A2190 20651018 10.3174/ajnr.A2190 PMC7965544

[71] Takai K, Endo T, Fujimoto S (2024) Angiographic challenges of spinal dural and epidural arteriovenous fistulas: report on 45 cases. Neuroradiology 66:279–286. 10.1007/s00234-023-03227-5 37792087 10.1007/s00234-023-03227-5

[72] Vargas MI, Gariani J, Sztajzel R et al (2015) Spinal cord ischemia: practical imaging tips, pearls, and pitfalls. AJNR Am J Neuroradiol 36:825. 10.3174/AJNR.A4118 25324492 10.3174/ajnr.A4118 PMC7990611

[73] Masson C, Pruvo JP, Meder JF et al (2004) Spinal cord infarction: clinical and magnetic resonance imaging findings and short term outcome. J Neurol Neurosurg Psychiatry 75:1431–1435. 10.1136/JNNP.2003.031724 15377691 10.1136/jnnp.2003.031724 PMC1738740

[74] Taterra D, Skinningsrud B, Pękala PA et al (2019) Artery of Adamkiewicz: a meta-analysis of anatomical characteristics. Neuroradiology 61:869. 10.1007/S00234-019-02207-Y 31030251 10.1007/s00234-019-02207-y PMC6620248

[75] Thurnher MM, Bammer R, Diffusion-weighted MR (2006) imaging (DWI) in spinal cord ischemia. Neuroradiology 48:795–801. 10.1007/S00234-006-0130-Z/METRICS 16977443 10.1007/s00234-006-0130-z

[76] Zalewski NL, Rabinstein AA, Krecke KN et al (2019) Characteristics of spontaneous spinal cord infarction and proposed diagnostic criteria. JAMA Neurol 76:56–63. 10.1001/jamaneurol.2018.2734 30264146 10.1001/jamaneurol.2018.2734 PMC6440254

[77] Faig J, Busse O, Salbeck R (1998) Vertebral body infarction as a confirmatory sign of spinal cord ischemic stroke: Report of three cases and review of the literature. Stroke 29:239–243. 10.1161/01.STR.29.1.239/ASSET/064C4739-35F9-4B7B-B694-6DF800574C88/ASSETS/GRAPHIC/HS0180002004.JPEG 9445357 10.1161/01.str.29.1.239

[78] Krings T, Mull M, Gilsbach JM, Thron A (2005) Spinal vascular malformations. Eur Radiol 15:267–278. 10.1007/s00330-004-2510-2 15538580 10.1007/s00330-004-2510-2

[79] Suh SH, Chung TS, Lee SK, Cho YE, Kim KS (2012) Ventriculus terminalis in adults: unusual magnetic resonance imaging features and review of the literature. Korean J Radiol 13:557. 10.3348/KJR.2012.13.5.557 22977322 10.3348/kjr.2012.13.5.557 PMC3435852

[80] Unsinn KM, Geley T, Freund MC, Gassner I (2000) US of the spinal cord in newborns: spectrum of normal findings, variants, congenital anomalies, and acquired diseases. Radiographics 20:923–938. 10.1148/RADIOGRAPHICS.20.4.G00JL06923 10.1148/radiographics.20.4.g00jl06923 10903684

[81] Hunsaker P, Gupta K, Otto N, Epelman MJ, Chandra T (2023) Developmental abnormalities of the pediatric spine: a review of the correlation between ultrasound and MRI findings. Cureus. 10.7759/CUREUS.44580 10.7759/cureus.44580 PMC10545393 37790066

[82] Jiblawi A, Chanbour H, Tayba A, Khayat H, Jiblawi K (2021) MRI characteristics of split cord malformation. Cureus. 10.7759/CUREUS.18328 10.7759/cureus.18328 PMC8553275 34725591

[83] Huang SL, He XJ, Xiang L, Yuan GL, Ning N, Lan BS (2014) CT and MRI features of patients with diastematomyelia. Spinal Cord 52:689–692. 10.1038/sc.2014.68 24796446 10.1038/sc.2014.68

[84] Gbadamosi WA, Daftari A, Szilagyi S (2022) Focal diastematomyelia in an adult: a case report. Cureus 14:e26081. 10.7759/cureus.26081 35875309 10.7759/cureus.26081 PMC9295302

[85] Kobayashi S, Uchida K, Takeno K et al (2006) Imaging of cauda equina edema in lumbar canal stenosis by using gadolinium-enhanced MR imaging: experimental constriction injury. AJNR Am J Neuroradiol 27:346 16484408 PMC8148809

[86] Kobayashi S, Yoshizawa H, Yamada S (2004) Pathology of lumbar nerve root compression Part 1: intraradicular inflammatory changes induced by mechanical compression. J Orthop Res 22:170–179. 10.1016/S0736-0266(03)00131-1 14656677 10.1016/S0736-0266(03)00131-1

