# Supplementary material for: Pathology of the conus medullaris and cauda equina. Beyond the usual suspects
Source: Insights Imaging. 2025 Oct 25;16:225. doi: 10.1186/s13244-025-02117-z (PMC12553641; doi:10.1186/s13244-025-02117-z)

Pathology of The Conus Medullaris and Cauda Equina. Beyond the usual Suspects.

ELECTRONIC SUPPLEMENTARY MATERIAL

Figure S1. 68-year-old patient with advanced breast cancer. A and B. Abnormal thick nodular T2 hypointense and enhancing lesions of the conus (blue arrows), with numerous multilevel nodular confluent enhancing lesion along the nerve roots of the cauda equina (yellows arrows). C. Leptomeningeal enhancement in the posterior fossa (red arrows).

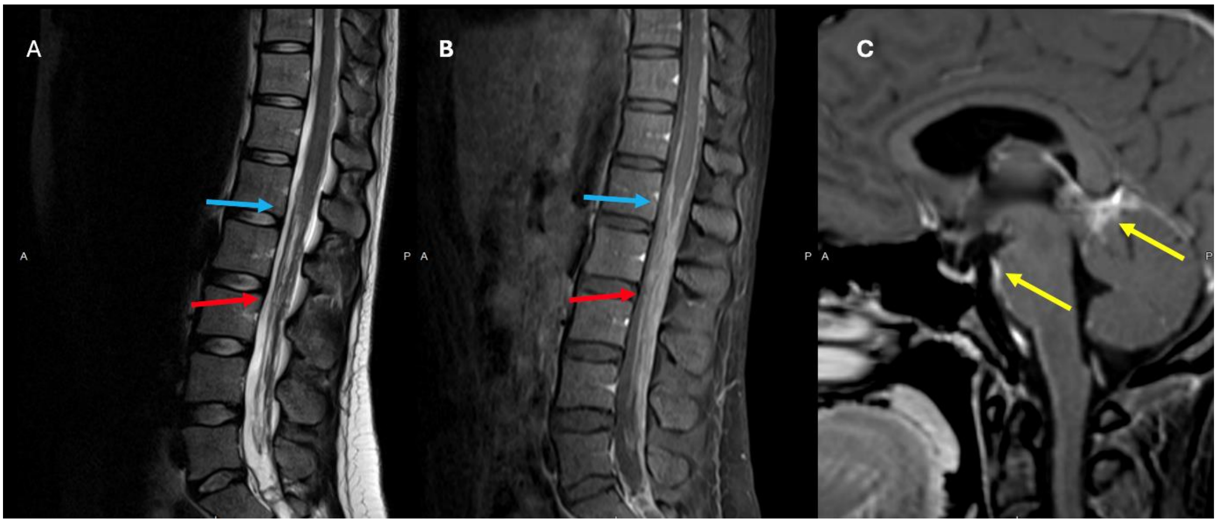

Figure S2. 57-year-old male patient with sepsis after posterior decompression surgery. A. Sagittal T2W image showing postsurgical changes from L4-L5 posterior decompression. B (Sagittal T1W) and C (Sagittal T1 postcontrast) showing enhancement of the cauda equina and surface of the conus (yellow arrows), as well as along the surgical bed (red arrows).

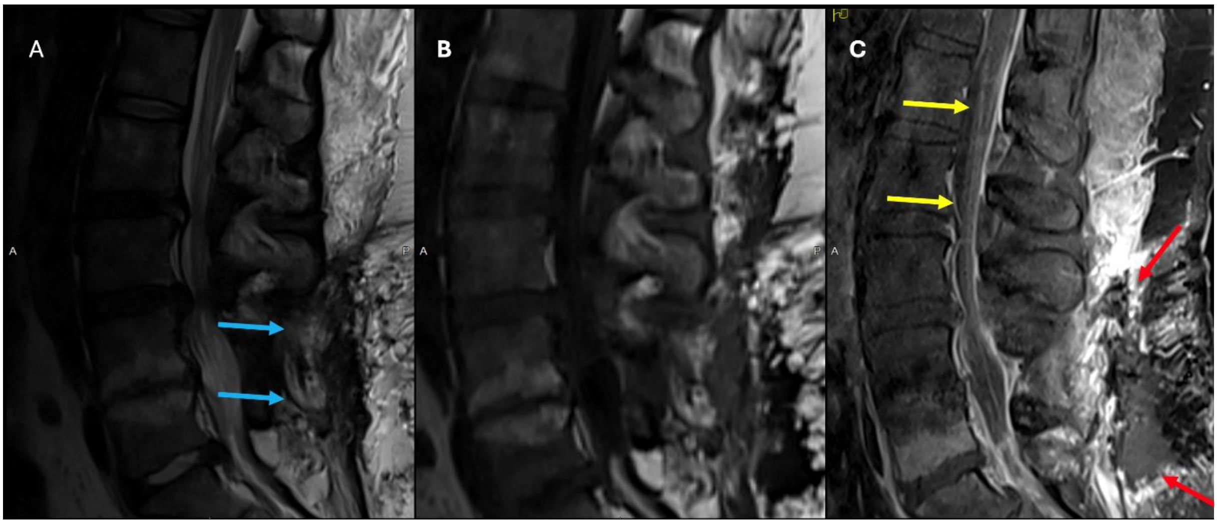

Figure S3. 66-year-old female who presented with history of headache, neck pain and nausea. Sagittal T1 postcontrast images showing dural thickening and enhancement in the cervical spine (A, blue arrow), Cauda equina and conus medullaris (B, yellow arrows), as well as in the prepontine cisterna and cerebellar leptomeninges (C, red arrows).

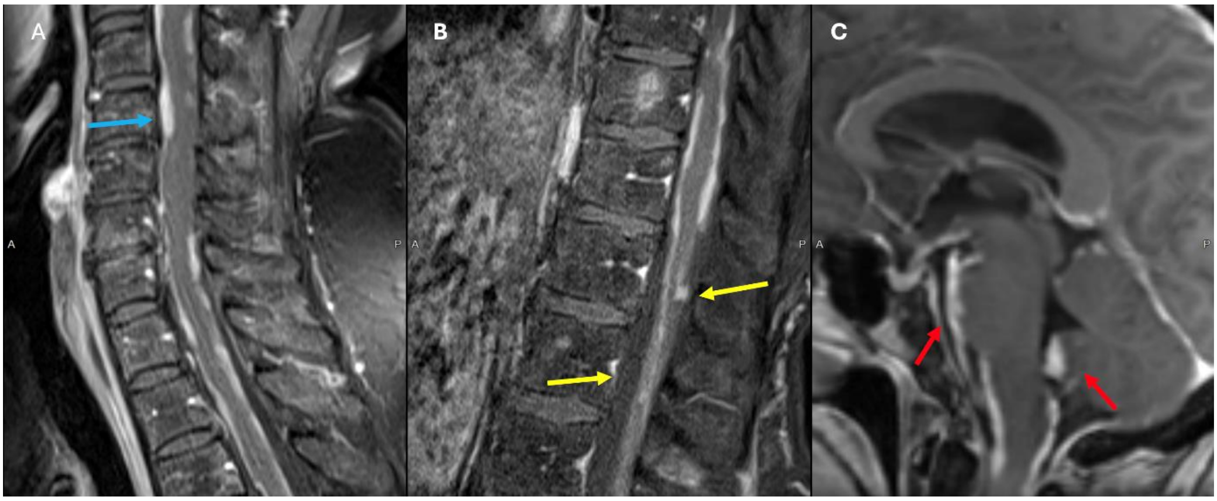

Figure S4. 30-year-old male with history of schizophrenia presenting with 2-3 months of inability to initiate swallowing and 2-3 months of gait clumsiness. A. Axial postcontrast image showing diffuse enhancement of the nerve roots surrounding the conus medullaris. B. Axial T2 image of the lumbar spine showing thickening of the cauda equina nerve roots (yellow arrow). C. Sagittal T1 postcontrast showing diffuse thickening and enhancement of the cauda equina nerve roots (red arrow).

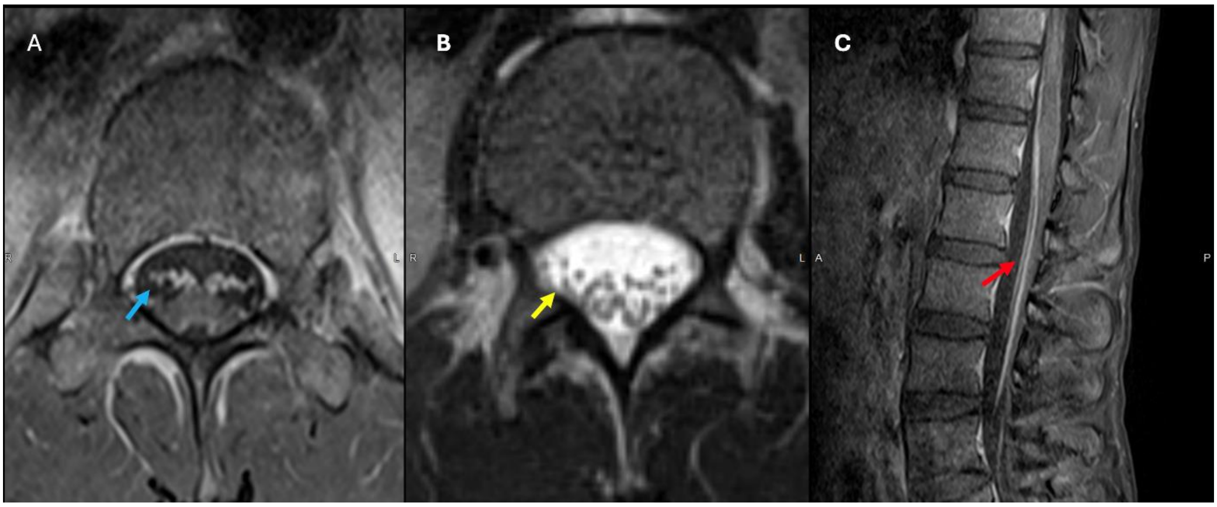

Figure S5. 49-year-old female who presented with back pain. A. Dilatation of central canal of the spinal cord is seen in conus medullaris (blue arrow). B (sagittal T2) and C (axial T2) showing cystic enlargement and hypersignal of the conus medullaris (yellow arrows).

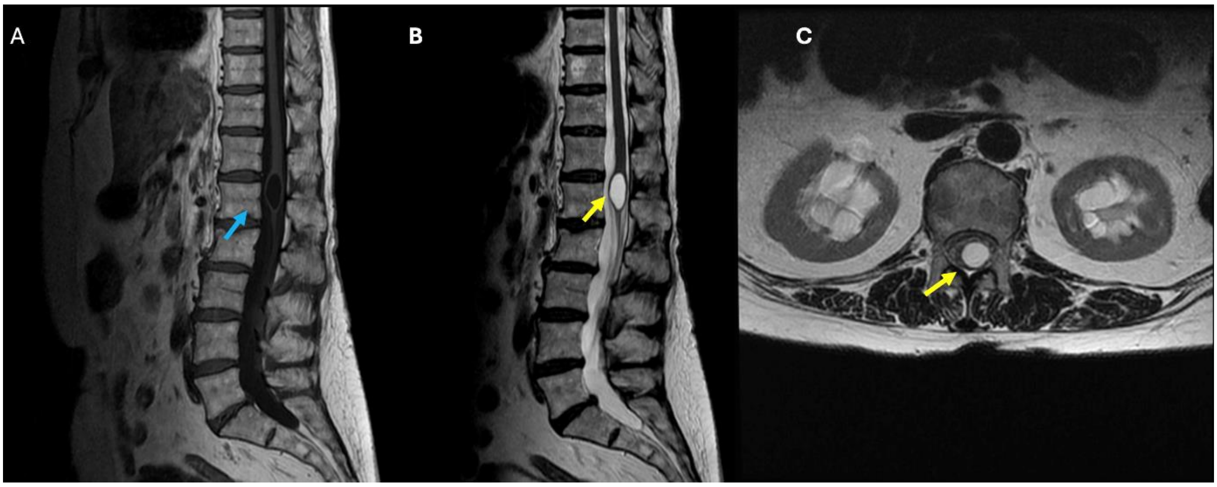

Figure S6. 82-year-old male who presented with back pain. A. Sagittal T1 postcontrast showing cauda equina enhancement along the anterior aspect of the conus medullaris (blue arrow). B. Sagittal T2 showing focal spinal canal stenosis at L1-L2 (yellow arrow). C. Axial T1 postcontrast showing anterior nerve root enhancement at L2-L3.

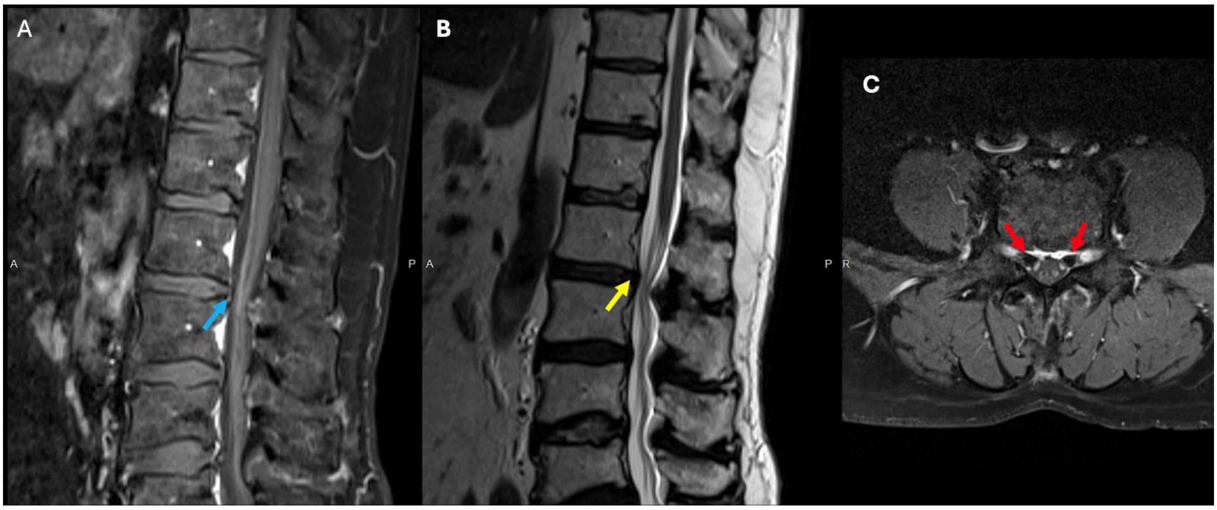

Supplement: Supplementary file 1 — ELECTRONIC SUPPLEMENTARY MATERIAL [file 13244_2025_2117_MOESM1_ESM.pdf]
